# Targeting the Roots of Kidney Disease: Systematic Review of the Therapies Targeting the Complement System

**DOI:** 10.3390/medicina61071205

**Published:** 2025-07-01

**Authors:** Maja Roman, Michał Nowicki

**Affiliations:** Department of Nephrology, Hypertension, Transplantation and Internal Medicine, Central University Hospital, Medical University of Lodz, Pomorska 251, 92-213 Lodz, Poland; maja.roman@stud.umed.lodz.pl

**Keywords:** perspective, complement system, kidney diseases, complement inhibition, targeted therapy

## Abstract

*Background/Objectives*: The field of nephrology is increasingly embracing advanced treatments and clinical trials that focus on inhibiting specific components of the complement cascade, a key driver in complement-mediated kidney diseases. *Materials and Methods*: This review aims to summarize innovative therapies targeting various pathways, including the inhibition of the terminal part of the complement pathway (mainly C5), the alternative pathway (factor B inhibitors), and the lectin pathway (MASP inhibitors. C5 inhibitors play a critical role in preventing the formation of the membrane attack complex (MAC), offering effective solutions for conditions like atypical hemolytic uremic syndrome (aHUS) and paroxysmal nocturnal hemoglobinuria (PNH). Meanwhile, avacopan, a C5a receptor antagonist, addresses ANCA-associated vasculitis (AAV) by mitigating inflammation and enabling reduced reliance on corticosteroids. Similarly, narsoplimab, which inhibits MASP-2, targets the lectin pathway implicated in conditions such as aHUS. Iptacopan, a factor B inhibitor, focuses on the alternative pathway and demonstrates efficacy in managing C3 glomerulopathy (C3G). *Results*: A systematic review of complement-targeted therapies was conducted, analysing studies from 2013 to 2023 that address unmet medical needs in primary and secondary glomerular diseases. *Conclusions*: Our systematic review of complement-targeted therapies shows that these tailored and innovative treatments may specifically address unmet medical needs in primary and secondary glomerular diseases.

## 1. Introduction

The complement system plays a crucial role as a component of innate immunity required for the detection, removal, and clearance of pathogens, immune complexes, and apoptotic cells. It consists of over 30 proteins, including enzymes, regulators, and receptors, and works within multiple cascades [1,2,3]. The activation of complements can occur in three principal processes: the classic pathway, which is induced by antigen-antibody complexes; a lectin pathway, which is initiated by pathogen-associated molecular patterns (PAMPs); and an alternative pathway, which is caused by PAMPs and spontaneous activation on the surfaces of microbes. All of these pathways converge at C3, which is a central protein leading to the formation of the membrane attack complex (MAC) that lyses target cells [1].

Since the glomeruli have a very extensive vascular bed, they are easily damaged when the complement system is affected. Such problems are the key to the understanding of diseases such as atypical hemolytic urea syndrome (aHUS), C3 glomerulopathy (C3G), transplant-associated thrombotic microangiopathy (TA-TMA), and IgA nephropathy (IgAN). In these disorders, a dysregulated complement cascade persists as a source of chronic inflammation that gradually erodes the glomerular structures and compromises kidney function overall [1,2].

Enhanced complement activation enhances immune reactions, thus leading to the formation of immune complexes and the enhancement of glomerular tissue destruction. This cascade of damage leads to kidney scars and increases the chances of developing end-stage renal disease (ESRD). The search for solutions to complement-related kidney injuries is complex because any treatment must target the cause while preserving the body’s protective immune system. Chronic complement activation exacerbates inflammation, promotes the deposition of the immune complex, and accelerates glomerular destruction, contributing to renal fibrosis and ESRD. Addressing complement-mediated kidney damage remains a major therapeutic challenge, requiring targeted therapies that precisely modulate complement activity [1].

**Figure 1:**
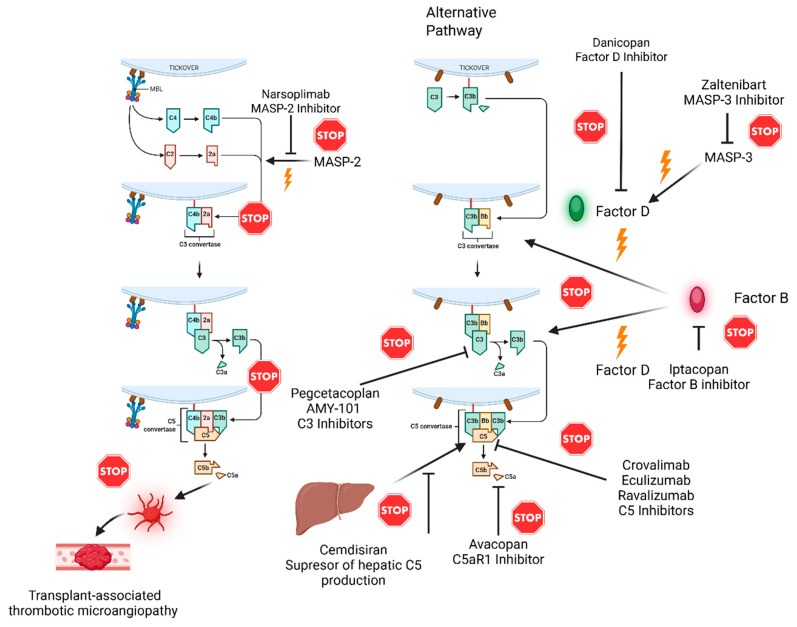
Cartoon showing targets of the action of different complement inhibitors in clinical use or being investigated in clinical trials.

The basic mechanisms of the action of drugs in the complement system are presented in Figure 1.

## 2. Sites of Action of Already Approved Drugs and Candidate Molecules in the Complement System

**Lectin Pathway: MASP-2 and MASP-3**

The lectin pathway is a major contributor to the complement system involved in innate immune response and inflammation. Two key proteases involved in this process are Mannan-binding protein-associated serine protease 2 (MASP-2) and Mannan-binding protein-associated serine protease 3 (MASP-3), which participate in distinct but related functions. The binding of MASP-2 to C4 and C2 activates C4, converting it to C4b, and C2, converting it to C2a, thereby generating C3 convertase and amplifying complement responses. In contrast, MASP-3 regulates an alternative pathway by activating factor D, a key enzyme necessary for its progression. The dysregulation of these proteases can lead to complement-mediated kidney damage and systemic inflammation [1].

Zaltenibart (OMS906) is an MASP-3 inhibitor used in complement-mediated diseases. It is being investigated for its ability to inhibit MASP-3 activity [4,5].

AMY-101 is another candidate drug that has promising beneficial effects and is being developed as a C3 inhibitor for inflammatory diseases, and it may be a significant step forward in complement-targeted therapies [6,7,8].

Danicopan is an oral factor D inhibitor that has been shown to reduce complement deposition and damage [9,10].

These agents are still undergoing clinical evaluation and have not yet received regulatory approval. Their precise role in future therapies will depend on further trial outcomes.

**Approved and Late-Stage Investigational Drugs**

**C3**

The C3 molecule is important for the complement cascade as it bridges the classical, alternative, and lectin pathways. Once it is cleaved, C3 gives rise to two active fragments: C3a, which triggers inflammation, and C3b, which acts to opsonize targets and induce the formation of C3 convertase. C3 dysregulation may lead to the deposition of immune complexes and kidney damage and is, therefore, considered a major target in complement-mediated diseases [6].

Pegcetacoplan is an anti-C3 agent that has been approved for the treatment of paroxysmal nocturnal hemoglobinuria (PNH). It inhibits the conversion of C3 to convertase, thereby preventing complement activation [6].

Clinical trials have shown that pegcetacoplan reduces proteinuria and stabilizes renal function in C3G [7].

**C5**

Eculizumab and its long-acting successor, ravulizumab are the latest C5 inhibitors. Ravulizumab allows less frequent dosing (every 8 weeks instead of every 2 weeks) compared to eculizumab, offering better patient compliance [11,12].

Crovalimab, another C5 inhibitor, prevents C5 cleavage, thereby blocking the formation of the pro-inflammatory mediator C5a and MAC, which exacerbates tissue damage. This action prevents C5a and MAC formation. Uncontrolled MAC formation seems to play a crucial role in the pathogenesis of target organ and tissue damage in several complement-related diseases [12].

Both eculizumab and ravulizumab have proven effective, reducing the need for dialysis and improving outcomes [11,13].

Eculizumab-aeeb is a monoclonal antibody that specifically binds to the complement protein C5, inhibiting its cleavage to C5a and C5b. This drug is the first approved interchangeable biosimilar to eculizumab [14].

**RNA-Based Therapy Targeting C5**

Cemdisiran is a conjugated small-interference RNA-based therapeutic that suppresses liver production of complement C5 and prevents the assembly of a membrane-attacking complex without broadly affecting complement activity. The high specificity of this approach minimizes the risk of infections observed with broader complement inhibition [15].

**Factor B**

Factor B is essential for C3 convertase formation in the alternative pathway. Blocking factor B reduces complement activation through this pathway without affecting the classical and lectin pathways, offering a targeted approach for complement inhibition. Iptacopan (LNP023) is an oral factor B inhibitor. It has shown promising results in reducing complement activation and deposition [16].

**C5a and C5aR**

C5a is a potent inflammatory mediator that interacts with the C5a receptor (C5aR) to recruit immune cells and amplify tissue damage. Targeting this pathway mitigates inflammation without disrupting the formation of MAC. Avacopan is an oral, selective C5aR inhibitor that mitigates inflammation without disrupting the formation of a MAC [11,17].

**Complement Regulatory Proteins: Factor H and Factor I**

Deficiencies or mutations in FH and FI disrupt complement regulation, leading to uncontrolled activation and subsequent tissue damage. Therapies targeting FH and FI aim to restore regulation and inhibit alternative pathway overactivation [18].

APL-2 (pegcetacoplan) stabilizes FH and FI, managing classical, lectin, and alternative pathway dysregulation [19].

A comparison of the mechanisms of action of drugs that inhibit the activation of the complement cascade is presented in Table 1.

## 3. Materials and Methods

A systematic search in PubMed and ClinicalTrials.gov was conducted to identify relevant studies on complement-targeted therapies for kidney disease. The search strategy was designed to capture the latest and most relevant clinical trials and peer-reviewed articles on this topic. The following search terms and Boolean operators were used: “complement inhibitors” OR “C5 inhibitor” OR “C3 inhibitor” OR “factor B inhibitor” OR “MASP inhibitor” OR “complement-targeted therapy” AND “kidney disease” OR “glomerulopathy” OR “aHUS” OR “PNH” OR “IgA nephropathy” OR “C3G” OR “transplant-associated thrombotic microangiopathy” OR “ANCA-associated vasculitis” AND “clinical trial” OR “Phase 1” OR “Phase 2” OR “Phase 3.” The searches were limited to English-language studies in human subjects. Only those studies with accessible full texts were included.

Studies were chosen according to pre-defined inclusion and exclusion criteria. The inclusion was for randomized controlled trials and observational studies that measured complement inhibitors, Phase 1 to 3 trials where Phase 1 trials were complete and had substantial clinical outcomes, and studies where the focus was on complement-mediated kidney diseases such as aHUS, C3G, IgAN, PNH, and TA-TMA and studies from the last 10 years that were published for the most current review. Exclusions from the analysis were in vitro studies and animal model studies, Phase 1 trials that were ongoing or had no results published, narrative reviews without systematic analysis, editorials and case reports, and studies that had insufficient information on outcomes or methodology.

The study selection process followed the Preferred Reporting Items for Systematic Reviews and Meta-Analyses (PRISMA) guidelines [25]. PubMed and ClinicalTrials.gov were searched using predetermined search terms to identify articles. Titles and abstracts were screened, and the duplicates were removed. Full papers were then screened against the inclusion and exclusion criteria. Studies meeting all the eligibility criteria were included in the systematic review.

No exclusion was made based on demographic factors like age, sex, or ethnicity since target-of-complement therapies are a newly evolving and highly innovative area of research, with numerous studies ongoing. Due to the paucity of long-term clinical data available, a wide inclusion strategy was adhered to to include all the information pertinent to the question without limiting study populations based on demographic data. 

**Inclusion criteria:**

Studies investigating complement-targeted therapies in complement-mediated kidney diseases, including paroxysmal nocturnal hemoglobinuria, atypical hemolytic uremic syndrome, C3 glomerulopathy, IgA nephropathy, transplant-associated thrombotic microangiopathy, and ANCA-associated vasculitis.

Randomized controlled trials and observational clinical studies.

Phase 1 (completed with published outcomes), Phase 2, and Phase 3 trials.

Studies published in peer-reviewed journals within the last 10 years.

Reporting of relevant clinical outcomes related to efficacy, safety, or kidney function.

No restriction on demographic variables (age, sex, ethnicity).

**Exclusion criteria:**

In vitro or animal studies.

Ongoing or incomplete Phase 1 trials without published results.

Narrative reviews, opinion articles, editorials.

Studies lacking adequate methodological detail or outcome data.

Duplicate publications, interim analyses, or reports without final results.

Articles not published in English.

**Figure 2:**
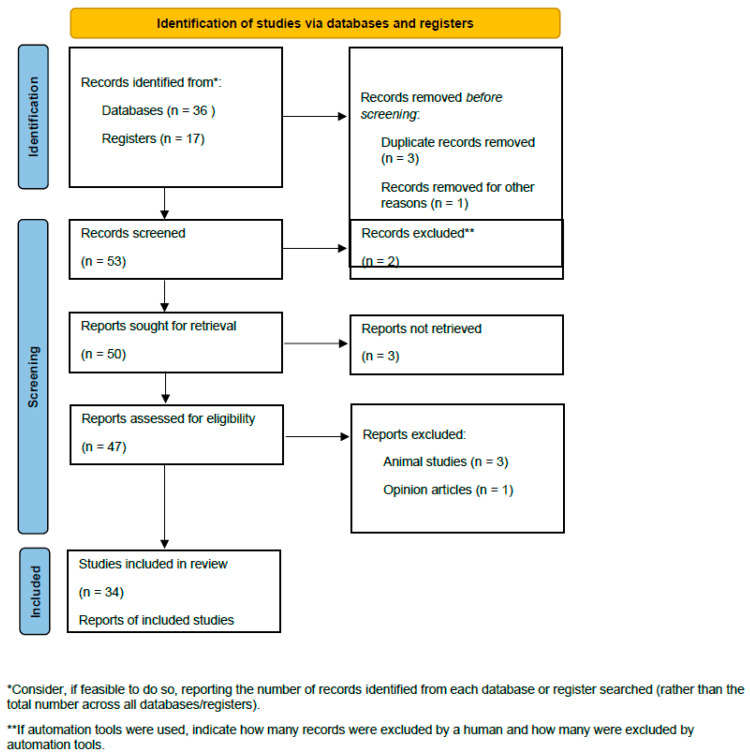
PRISMA 2020 flow diagram for new systematic reviews which included searches of databases and registers only [25].

The selection process of studies is illustrated in Figure 2.

Of the clinical trials that were assessed, four had a low risk of bias, two had a moderate risk of bias, and two had a moderate to high risk of bias. For one of the trials, the overall risk of bias could not be ascertained since the trial was ongoing, and no full data were available. The assessment was performed using the RoB 2 tool for randomized trials and the ROBINS-I tool for non-randomized trials. Nine clinical trials were included in this systematic review overall. Six trials were assessed depending on the study design, either using the RoB 2 tool or using the ROBINS-I tool (three trials).

A meta-analysis was not feasible at this stage due to the heterogeneity of the study designs, endpoints, and outcome reporting in the currently available clinical trials. A meta-analysis is planned as a subsequent step following the completion of ongoing studies.

The protocol for this systematic review was registered in the PROSPERO database under registration number 1052717.

## 4. Complement-Related Kidney Diseases for Which Drugs Are Already Available or Are in Late-Phase Clinical Trials

**Paroxysmal nocturnal hemoglobinuria (PNH)**

Paroxysmal nocturnal hemoglobinuria is caused by complement dysregulation, leading to intravascular hemolysis, thrombosis, and impaired bone marrow function. Treatments include eculizumab, ravulizumab, crovalimab, and pegcetacoplan [5,6]. Crovalimab was based on trials that demonstrated reducing transfusion requirements and improving hemoglobin levels in PNH patients. In the Phase 3 trial COMMODORE 2, crovalimab maintained disease control in 92.9% of patients, as characterized by hemolysis control (central LDH ≤ 1.5 × ULN), consistent with 93.7% observed in eculizumab-treated patients. Transfusion avoidance with crovalimab was achieved in 79.5% of patients compared to a rate for patients treated with eculizumab of 78.4%. These results confirm the non-inferiority of crovalimab to eculizumab in the treatment of PNH, with an added advantage of a more convenient dosing schedule, allowing for subcutaneous administration every four weeks compared to intravenous dosing every two weeks for eculizumab [12].

Most of the cited trials for PNH therapies are Phase 3 interventional studies involving adult cohorts. No data were provided on specimen types or biomarker analysis methods.

**Atypical hemolytic uremic syndrome (aHUS)**

Atypical hemolytic uremic syndrome is an inherited or acquired disease caused by dysregulation of the complement system that leads to hemolytic microangiopathic anemia, thrombocytopenia, and progressive kidney or other organ damage [26,27]. The approved targeted therapies for this disease treatment include the C5 inhibitors eculizumab and ravulizumab, but several other candidate drugs are currently being investigated in clinical trials.

Eculizumab, the first approved therapy for aHUS, demonstrated efficacy in reducing complement-mediated kidney damage. Long-term treatment with eculizumab reduced the risk of renal failure in patients with aHUS from 50% to 6–15% within one year of treatment [28]. It is worth noting that the U.S. Food and Drug Administration (FDA) has approved the first biosimilar to eculizumab [14].

Ravulizumab, a long-acting C5 inhibitor, offers similar efficacy to eculizumab but with a less frequent dosing schedule, administered every eight weeks. The results of a clinical trial [22] suggest that extended dosing intervals improve patient adherence and overall quality of life.

Crovalimab has been under development for aHUS and is being studied in several Phase 3 trials (COMMUTE-a, NCT04861259, and COMMUTE-p, NCT04958265). This compound is expected to be similarly effective as the current products on the market, with an added advantage of less frequent subcutaneous dosing [29]. It remains to be proved whether the new treatment will provide an advantage in the management of the underlying complement dysregulation of aHUS, improving patient outcomes and quality of life [30].

It is worth adding that although plasma infusion and exchange have been widely used in the treatment of aHUS for decades as an initial approach, the availability of targeted therapies with C5 inhibitors has relegated them to solely supportive treatment [31].

These trials primarily involve both pediatric and adult cohorts and are in Phase 3. The study designs include multiple arms; however, no data were provided on specimen types or biomarker analysis methods.

**C3 glomerulopathy (C3G)**

C3 glomerulopathy is caused by the dysregulation of C3 activation, which results in progressive kidney damage due to the deposition of immune complexes in the glomeruli. Investigational treatments include pegcetacoplan and iptacopan, which have shown some promise in Phase 2 clinical trials. The use of iptacopan is expected to minimize the use of aggressive immunosuppression and repeated hospitalization, providing an improved quality of life for patients while limiting disease progression to ESRD [32,33].

Another novel agent is the therapeutic peptide AMY-101, which is the first drug to be granted an orphan drug status by the European Medicines Agency (EMA) and the U.S. FDA for the indication of C3G. AMY-101 acts to limit inflammation and renal injury [34].

Most trials for C3G are Phase 2 and 3, but again, the protocols do not mention which types of biological specimens were collected.

**IgA nephropathy (IgAN)**

IgA nephropathy is a renal disease caused by the deposition of IgA-containing immune complexes in glomeruli. This subsequently produces inflammation, which can lead to progressive kidney damage. Treatments currently under investigation include iptacopan, a factor B inhibitor, which has shown promising efficacy in reducing proteinuria and preserving renal function. In the APPLAUSE-IgAN trial (NCT04578834), iptacopan showed a 38.3% reduction in proteinuria at 9 months compared to the placebo, with delayed disease progression [35].

Cemdisiran, in Phase 1 trials, demonstrated safety and effective C5 suppression [15].

Avacopan, in an open-label pilot study among IgAN patients with persistent proteinuria despite maximal supportive care, has shown renal anti-inflammatory activity, with a 30% reduction of urinary monocyte chemoattractant protein-1 to creatinine ratio by week 8 [36].

The IgAN study with the MASP-2 inhibitor narsoplimab, ARTEMIS-IgAN, was prematurely terminated in 2023 due to a lack of efficacy, and, therefore, work on this molecule for the treatment of IgAN nephropathy was discontinued [37].

The available clinical trials for IgAN therapies vary from Phase 1 to 3. No data were provided on specimen types or biomarker analysis methods.

**Transplant-associated thrombotic microangiopathy (TA-TMA)**

Transplant-associated thrombotic microangiopathy is a complement dysregulation-mediated vascular injury that may lead to graft loss following transplantation. Investigational treatments include narsoplimab, a MASP-2 inhibitor, which has shown promise in reducing complement-mediated vascular injury [3,13,38].

In a Phase 3 study (NCT02949128), ravulizumab achieved a complete thrombotic microangiopathy response in 61% to 71% of patients, depending on the baseline dialysis status, and provided sustained renal protection with improvement in quality of life. In a Phase 3 study, ALXN1210-aHUS-311, 53.6% of the patients fully responded to the TA-TMA induced by ravulizumab in up to 26 weeks. In addition, an improvement in renal function was observed for 68% of the patients, while dialysis weaning could be undertaken in 58% of those patients who were on dialysis at baseline [13].

Eculizumab and ravulizumab lead to improved renal outcomes in both adult and pediatric populations. This is, however, at the expense of an increased risk of invasive meningococcal infections, which is reduced by obligatory vaccination against meningococci, pneumococci, and Haemophilus influenzae type B. Additionally, amoxicillin is used orally for the prophylaxis against meningococci [13,22,39,40].

In clinical trials, narsoplimab was effective in improving markers of endothelial damage and overall clinical outcomes in patients with hematopoietic stem cell transplant-associated thrombotic microangiopathy. However, this agent has not yet received FDA or EMA approval for this indication. Further studies are needed [41].

The trials included both pediatric and adult populations. No data were provided on specimen types or biomarker analysis methods.

**ANCA-associated vasculitis (AAV)**

AAV is an inflammatory disease driven by complement activation [1], resulting in neutrophil recruitment and tissue damage that can affect multiple organs. Treatments for AAV include avacopan [17].

In a Phase 3 trial (NCT02994927), avacopan achieved a 72.3% remission rate at 26 weeks, demonstrating its effectiveness as a steroid-sparing therapy. This approach reduces corticosteroid-related side effects while improving clinical outcomes and patient quality of life [42].

The trial design for avacopan included control arms and well-defined endpoints. No data were provided on specimen types or biomarker analysis methods.

A summary of the information from ongoing clinical trials can be found in Table 2.

## 5. History of Approvals and Clinical Development of Complement-Targeting Therapies for Kidney Diseases

Eculizumab was approved by the FDA in March 2007 for PNH and in September 2011 for aHUS, but it has also been tested in C3G. Though Phase 2 trials began in 2013 (NCT01720184), it has not yet received approval for this indication [46,47]. Eculizumab was approved by the EMA in June 2007 for PNH and in July 2009 for aHUS [48].

Ravulizumab was approved by the FDA in December 2018 for PNH and in October 2019 for aHUS, based on a Phase 3 trial (NCT02949128) [49]. The EMA approved ravulizumab for PNH in July 2019 [48] and for aHUS in November 2019 [50].

The FDA also approved crovalimab in June 2024 for PNH, and the EMA approved it in September 2024 for PNH; Phase 3 trials are currently ongoing for aHUS (NCT04434092) and lupus nephritis (NCT04958265) [51,52,53].

The FDA approved avacopan for AAV, including GPA and MPA, in October 2021; the EMA approved it in January 2022 [54,55].

Iptacopan was approved by the FDA in December 2023 and by the EMA in March 2024 for PNH, with Phase 3 trials still continuing for C3G (NCT04817618), IgA nephropathy (NCT04817618), and aHUS (NCT05795140) [56,57,58]. The Phase 3 ARTEMIS-IgAN trial (NCT03608033) for narsoplimab in IgA nephropathy concluded in 2023 and was deemed a failure [59].

FDA Fast Track status was assigned for aHUS, and a Biologics License Application for TA-TMA has been filed, although it has not been approved to date [60].

Cemdisiran completed its Phase 2 studies in IgA nephropathy in 2022 and, since 2021, has FDA Orphan Drug Designation status; Phase 2 studies are currently actively recruiting for aHUS (NCT03841448) and PNH (NCT03999840) [15,61]. A formal recommendation has not yet been issued by the EMA on cemdisiran.

Currently, there are clinical trials in place with AMY-101, including a Phase 2 for C3G, NCT05025943, and completed Phase 1 trials for PNH, NCT04670060 [62,63,64].

APL-2 was approved by the FDA in May 2021 for PNH and is currently in Phase 3 trials for C3G (NCT03500315) and IgA nephropathy (NCT04577807) [34,65]. The EMA approved APL-2 for PNH in July 2021 [65,66].

Zaltenibart completed Phase 2 trials for PNH with promising results (NCT04293262) and is planning Phase 3 trials for C3G. The FDA has specified it with Orphan Drug and Rare Pediatric Disease designations for C3G [67,68,69].

The EMA has also approved several new complement system inhibitors. Ravulizumab was recommended for marketing authorization in the European Union for PNH and aHUS, providing a longer-acting alternative to eculizumab [48]. EMA approvals for avacopan marked a major turning point in managing ANCA-associated vasculitis, reducing reliance on high-dose glucocorticoids [53]. More recently, in March 2024, the EMA approved iptacopan for PNH, continuing to expand the options for treating complement-mediated diseases [54,56]. A comparison of drug side effects and long-term data is provided in Table 3.

## 6. Limitations

This systematic review has several limitations that should be considered when interpreting the findings. One of the main limitations is the publication bias, in which studies that have positive or favorable results are more likely to be published compared to studies with negative or inconclusive results. This can lead to overestimation of the effectiveness of complement-targeted therapies. In addition, the majority of the studies covered were sponsored by pharmaceutical industries, and this may result in bias in the study design, reporting, and interpretation of the results.

Among the limitations is the heterogeneity of the included studies, particularly in terms of the study populations, treatment regimens, follow-up durations, and outcome measures. The differences in the dosing regimens, endpoints, and patient populations complicate a direct comparison and may potentially limit the generalizability of the findings. Furthermore, while randomized controlled trials are a high-quality source of evidence, some of the included studies were observational or based on early-phase clinical trials, which are a lower level of evidence by design. Most of the included studies were industry-sponsored and lacked blinding, increasing the potential risk of bias.

One of the main limitations is the lack of long-term efficacy and safety data for most of these therapies. Follow-up periods were relatively short for most of the trials in this review, so it was not possible to ascertain the long-term outcomes of the treatments, late-emerging adverse effects, and durability of the treatment effects. This is particularly applicable to chronic kidney diseases, where the progression of the disease may occur over many years.

Furthermore, this review did not exclude studies by age, sex, or ethnicity since complement-targeted therapies are a comparatively new and highly innovative area of research. However, due to the relatively small sample sizes of the clinical trials, subgroup analyses stratified for demographic factors were generally limited, such that potential differences in treatment responses in various patient groups are unknown.

## 7. Future Research Directions

Subsequent research on complement-targeted kidney disease treatments must consider a few key areas to maximize clinical benefit and therapeutic value. Foremost among these is the need for robust long-term efficacy and safety surveillance. Since most existing trials have short follow-up, subsequent studies must determine the durability of the treatment benefit, late adverse effects, and impact on the course of chronic kidney disease.

Further research is needed to identify predictive biomarkers of response to complement-targeted therapies. Several candidate biomarkers, such as plasma levels of C3, C5a, soluble C5b-9, LDH, or proteinuria, can potentially be used to monitor treatment response. In general, the specimen types are serum, plasma, and urine, with kidney biopsies rarely being used to assess complement deposition. The absence of validated companion diagnostics currently limits the personalization of complement inhibition, underscoring the need for prospective studies integrating molecular profiling and functional complement assays.

Promising study medications such as AMY-101 and iptacopan have shown encouraging outcomes in phase trials; however, their efficacy in larger and more heterogeneous populations must be established. Randomized controlled trials that directly compare the available C5 inhibitors are needed to identify the best and safest treatment strategies. In parallel, mechanistic studies must determine whether an upstream complement blockade (e.g., C3 level) adds a synergy or value over inhibition downstream. Despite promising results, the heterogeneity of the trial design and a limited sample size exclude a meaningful comparison. In addition, the discovery of predictive biomarkers to guide therapy duration and enable patient stratification is important, such that early recognition of those who are most likely to benefit can be enabled. Future trials would similarly need to be designed to best optimize generalizability by recruiting underrepresented groups along age, ethnic, and comorbidity spectrums to determine potential differences in response to therapy.

Finally, health-economic analysis and real-world evidence evaluations must be integrated in future development to guide cost-effectiveness, accessibility, and application in clinical practice.

## 8. Conclusions

In summary, the complement system has been implicated in the pathogenesis of several renal diseases, and the list of complement-dependent diseases continues to grow due to continuous advances in the understanding of its role in the human body, well beyond its traditional protective function against infection. Over the past decade, significant progress has been made in the treatment of complement-dependent diseases, driven by existing unmet clinical needs and developments in drug manufacturing technologies. Although these are not cure-alls for diseases in nephrology, drugs targeting the complement system are expected to transform the treatment of a wide range of kidney diseases, from ultra-rare diseases, such as aHUS, to relatively common diseases, such as IgA nephropathy or several other primary glomerulopathies.

## Figures and Tables

**Table 1:** Mechanisms of action of complement inhibitors.

Drug	Target	Pathway	Mechanism
Pegcetacoplan [6,7]	C3	Terminal	Inhibits C3 cleavage, blocking complement activation and MAC formation [6,7].
AMY-101 [8]	C3	Terminal	Inhibits C3, reducing inflammation and preventing MAC formation [8].
Eculizumab [20,21]	C5	Terminal	Blocks cleavage into C5a and C5b, preventing MAC formation [20,21].
Ravulizumab [22]	C5	Terminal	Long-acting C5 inhibitor offering extended dosing intervals [22].
Crovalimab [12]	C5	Terminal	Subcutaneous formulation; prevents MAC formation [12].
Avacopan [13,23]	C5a receptor (C5aR1)	Alternative	Blocks C5a receptor on neutrophils, inhibiting inflammation [13,23].
Iptacopan [24]	Factor B	Alternative	Inhibits alternative pathway amplification by targeting the C3 convertase [24].
Narsoplimab [13,23]	MASP-2	Lectin	Prevents activation of the lectin pathway by inhibiting MASP-2 [13,23].
Cemdisiran [15]	C5 (gene level)	Terminal	RNA interference reduces C5 production in hepatocytes [13].
Biosimilars [14]	C5 (e.g., eculizumab-aeeb)	Terminal	Cost-effective alternatives with identical mechanisms [12].

**Table 2:** Clinical outcomes of recent trials.

Drug	Disease	Trial Phase	Outcome	NCT Number	Significance
Ravulizumab [43]	aHUS	Phase 3	61–90% TA-TMA response, improved renal function	NCT02949128	Extended dosing intervals improve adherence.
Avacopan [42]	AAV	Phase 3	72.3% remission at 26 weeks	NCT02994927	Steroid-sparing option reduces adverse effects.
Iptacopan [44]	C3G	Phase 3	Reduced proteinuria, delayed disease progression	NCT04817618	Oral therapy offers convenience.
Cemdisiran [45]	IgA Nephropathy (early stage)	Phase 1	Safe, effective C5 suppression	NCT03841448	First RNAi-based complement therapy.

**Table 3:** Comparison of mechanisms, safety profiles, and long-term data of complement system inhibitors.

Drugs	Diseases	Side Effects	Long-Term Data
Eculizumab	aHUS, PNH [6,11,21]	Risk of meningococcal infections [13,26]	Limited to 1 year efficacy [70]
Ravulizumab	aHUS, PNH [46,47]	Like eculizumab [13,23]	Well tolerated [71]; 93% of patients preferred ravulizumab to eculizumab [72]
Crovalimab	aHUS, PNH [12,27]	Like eculizumab [72,73]	Awaiting long-term outcomes
Iptacopan	PNH, C3G, IgAN [16,24,32,57]	Risk of severe infections [74]	Awaiting long-term outcomes
Pegcetacoplan	PNH, C3G [6,29]	Risk of severe infections [75]	Awaiting long-term outcomes
AMY-101	C3G [34]	Not available yet	Awaiting long-term outcomes
Avacopan	IgAN, C3G [36]	Allergy symptoms, digestive problems [76,77]	Awaiting long-term outcomes
Cemdisiran	aHUS, IgAN, PNH [15,45,66]	Sleep disorders, nausea, headache [78]	Awaiting long-term outcomes
Narsoplimab	TA-TMA [4]	Digestive problems, neutropenia, nausea [79]	Awaiting long-term outcomes
